# BRAIN FUNCTIONAL CONNECTIVITY IN OLDER ADULTS WITH MCI: DO FALLS MATTER?

**DOI:** 10.1093/geroni/igz038.1569

**Published:** 2019-11-08

**Authors:** Anna R Egbert, Chun Liang Hsu, Rachel Crockett, Teresa Liu-Ambrose

**Affiliations:** University of British Columbia, Vancouver, British Columbia, Canada

## Abstract

Older adults with mild cognitive impairment (MCI) are at an elevated risk of falls. We conducted a pilot longitudinal observational study to examine the natural course of brain intrinsic functional connectivity (FC) and cognitive function changes in association to falling history in older adults with MCI. 15 MCI participants (mean age 75.9, range 67-86) included 10 non-fallers and 5 fallers (minimum two falls in the previous 12 months with one in the last 6 months) from Metro Vancouver, BC, Canada. At study entry and 1-year follow-up, participants completed brain scanning session of structural MRI and resting state (RS) functional MRI, the Montreal Cognitive Assessment (MoCA), and the Mini Mental State Examination (MMSE). Results indicated an interaction between time (baseline vs. follow-up) and falls history on RS-FC in individuals with MCI (p<0.001). At 1-year follow-up, MCI non-fallers showed increased FC between frontal, parietal and occipital cortex (from baseline R=0.141 to follow-up R=0.321) and lack of decline on cognitive measures. Meanwhile, MCI fallers showed weakening of FC between those brain regions (from baseline R=0.314 to follow-up R=0.201) with simultaneous cognitive deterioration. Significant relationships between FC strength and cognitive status existed only at follow-up (R=0.525, p<0.05), suggesting that the triggered functional compensatory brain mechanisms in MCI non-fallers are not successfully executed in MCI fallers. Together, our pilot data suggest that older adults with MCI who fall show more advanced brain functional degradation with adjacent cognitive decline as compared to MCI individuals who do not fall.

